# Gene Expression Signatures Reveal Common Virus Infection Pathways in Target Tissues of Type 1 Diabetes, Hashimoto’s Thyroiditis, and Celiac Disease

**DOI:** 10.3389/fimmu.2022.891698

**Published:** 2022-06-20

**Authors:** Min Yin, Yan Zhang, Shanshan Liu, Juan Huang, Xia Li

**Affiliations:** ^1^National Clinical Research Center for Metabolic Diseases, Key Laboratory of Diabetes Immunology, Ministry of Education, Changsha, China; ^2^Department of Metabolism and Endocrinology, The Second Xiangya Hospital of Central South University, Changsha, China; ^3^Section of Endocrinology, Department of Internal Medicine, School of Medicine, Yale University, New Haven, CT, United States

**Keywords:** type 1 diabetes, Hashimoto’s thyroiditis, celiac disease, gene expression signatures, target tissues

## Abstract

Type 1 diabetes (T1D) patients are at heightened risk for other autoimmune disorders, particularly Hashimoto’s thyroiditis (HT) and celiac disease (CD). Recent evidence suggests that target tissues of autoimmune diseases engage in a harmful dialogue with the immune system. However, it is unclear whether shared mechanisms drive similar molecular signatures at the target tissues among T1D, HT, and CD. In our current study, microarray datasets were obtained and mined to identify gene signatures from disease-specific targeted tissues including the pancreas, thyroid, and intestine from individuals with T1D, HT, and CD, as well as their matched controls. Further, the threshold-free algorithm rank-rank hypergeometric overlap analysis (RRHO) was used to compare the genomic signatures of the target tissues of the three autoimmune diseases. Next, promising drugs that could potentially reverse the observed signatures in patients with two or more autoimmune disorders were identified using the cloud-based CLUE software platform. Finally, microarray data of auto-antibody positive individuals but not diagnosed with T1D and single cell sequencing data of patients with T1D and HT were used to validate the shared transcriptomic fingerprint. Our findings revealed significant common gene expression changes in target tissues of the three autoimmune diseases studied, many of which are associated with virus infections, including influenza A, human T-lymphotropic virus type 1, and herpes simplex infection. These findings support the importance of common environmental factors in the pathogenesis of T1D, HT, and CD.

## Introduction

Type 1 diabetes (T1D), with a worldwide increasing incidence, is characterized by the autoimmune destruction of pancreatic beta cells, resulting in life-long insulin dependency (1). Aside from islet autoimmunity, patients with T1D are at heightened risk for other autoimmune disorders, particularly Hashimoto’s thyroiditis (HT) and celiac disease (CD), both of which are organ-targeted autoimmune diseases (2–4), implying common risk factors and molecular mechanisms among these three autoimmune diseases.

Although the pathogenesis of autoimmune diseases is not completely understood, both environmental and genetic factors have been linked to its pathogenesis, which is marked by the development of specific autoantibodies and the existence of autoreactive T cells. Genetically, these three autoimmune diseases share similar genetic predispositions with similar human leukocyte antigen (HLA) susceptibility (5). However, with the rising prevalence of autoimmune diseases, common environmental risk factors for T1D, HT and CD should be emphasized. Whether shared environmental mechanisms drive identical molecular mechanisms at target tissues among T1D, HT, and CD is unclear.

Increasing evidence suggests that target tissues of autoimmune diseases engage in a harmful dialogue with the immune system instead of being innocent bystanders of immunological attacks (6). In this sense, a study focusing on different tissues of these autoimmune diseases could aid in identifying essential pathways that could be focused for therapy, such as repurposing drugs that are already in clinical use for other diseases. Based on this, we hypothesize that key environmental mechanisms may drive similar molecular signatures in T1D, HT, and CD at the target tissue level. To test this hypothesis, we obtained microarray datasets generated from disease-specific targeted tissues including the pancreas, thyroid, or intestine from individuals with T1D, HT, and CD respectively, along with their control subjects. These data were dug to identify similar and dissimilar transcriptomic signatures. Then, we searched for drugs that could potentially reverse the observed signatures in patients with two or more autoimmune disorders. Our findings revealed significantly similar transcriptomic signatures in the target tissues of these three autoimmune diseases studied, many of which are associated with virus infections, including influenza A, human T-lymphotropic virus type 1 (HTLV-1), and herpes simplex infection. These findings support the importance of common environmental factors in the pathogenesis of T1D, HT, and CD.

## Methods

### Microarray Data

To identify shared transcriptomic signatures in T1D, HT, and CD at the target tissue level, three gene expression profiles by array were selected from the Gene Expression Omnibus (GEO) database (http://www.ncbi.nlm.nih.gov/geo/). The T1D dataset GSE72492 (7) contained pancreas samples from 10 T1D patients, 6 auto-antibody positive individuals (AP) but not diagnosed with T1D and 7 normal controls (NC). In the HT dataset GSE138198 (8), data from 13 HT thyroid samples and three normal thyroid samples were used for analysis. The CD dataset GSE164883 (9) included intestinal tissues samples from 25 CD patients and 21 normal controls. Two CD patients were sampled twice and all 48 samples were used for further analysis. The HLA genotype of the T1D dataset and basic information of the three datasets were obtained from the corresponding original article and presented in the **Table 1 part**.

### Single Cell RNA-Seq Data and Processing

To validate our findings in the microarray data, we obtained single cell RNA-seq data (RRID: SCR_016202) from the Human Pancreas Analysis Program (HPAP-RRID: SCR_016202) Database (https://hpap.pmacs.upenn.edu) (10). CellRanger standard output files were downloaded containing data from islet samples of one patient with T1D and HT (HPAP-032) and one normal control (HPAP-039) matched for race and gender. Seruat 4.0 package in R was used to read the CellRanger standard output file for analysis. Cells are considered abnormal if: (i) gene number was <200 or >4000; (ii) over 25% of the detected genes were mitochondrial genes. Abnormal cells in all datasets were filtered out. The DEGs criteria were as follows using the Find All Markers function (Searut package): (i) logFC>0.585; (ii) *p*-value<0.05; (iii) min.pct>0.25. The GSEA enrichment analysis of DEGs of single cell RNA-seq was performed using OmicStudio tools (https://www.omicstudio.cn/tool).

### Differentially Expressed Genes Analysis

To identify the DEGs responsible for T1D, HT, and CD, the matrix files of three datasets were downloaded from GEO and normalized and annotated using the online tool NetworkAnalyst 3.0 (11) (https://www.networkanalyst.ca/). Probe IDs were transformed into official gene symbols. Probes with no gene symbol name were deleted. Multiple probes related to the same gene were deleted and summarized as the average value for further analysis. The limma package in R was used to perform DEGs analysis by comparing patients with normal controls. For DEGs identification, |log2 fold change (FC)| ≥ 0.585 and *p*-value <0.05 were considered statistically significant. An overlap between the three lists of genes was then performed and represented as a Venn diagram.

### Risk Genes Identification

To explore the association between DEGs and genetic variation polymorphism in target organs is related to genetic variation polymorphism, and to describe the influence of genetic factors in autoimmune diseases, the genome-wide association studies (GWAS) catalog (12) (www.ebi.ac.uk/gwas/; consulted December 2021) was used to identify the risk genes connected with each disorder. The risk genes were identified based on the following criteria: (i) T1D, HT, and CD as the disease assessed by the study; (ii) a *p-value* of < 1 × 10^−6^ for the lead single-nucleotide polymorphism (SNP). (iii) Choosing the recorded genes linked to the lead SNP described by the original study;(iv) assessing the expression of the recorded genes in the target tissue. An overlap between risk genes and DEGs in each disease and an overlap between the three lists of risk genes were represented as a Venn diagram.

### Rank-Rank Hypergeometric Overlap Analysis

To compare the transcriptomic signatures of the target tissues of T1D, HT, and CD, overlaps between the differential expression of two ranked lists were visualized and measured using online RRHO tools (https://systems.crump.ucla.edu/rankrank/index.php) (13). Briefly, all expressed genes from three microarray datasets were ranked according to fold change value. Then, these ranked lists were iteratively assessed for intersection. Finally, the results were visualized as a heatmap colored by the logarithmic transformation’s hypergeometric *p* value. The *p* value assessed the significance of overlapping genes at each rank threshold pair so that the highest point on the map identified the overlapping genes with the most statistical significance. Genes overlapping at this optimal rank threshold pair in all of the three probable pairing combinations were listed and assessed further for involvement in specific biological characteristics and signaling pathways.

### Functional Enrichment Analysis

To identify the biological function of DEGs of three microarray datasets and overlapping genes in RRHO analysis, enrichment analysis of Gene Ontology (GO) and Kyoto Encyclopedia of Genes and Genomes (KEGG) pathways was performed using the online tool Database for Annotation, Visualization and Integrated Discovery (DAVID) 6.8 (14) (https://david.ncifcrf.gov/). All KEGG terms with gene count ≥ 2 and *p*-values < 0.05 were visualized.

### Therapeutic Target Identification

To explore drugs or compounds that target the shared pathways in autoimmune diseases, we selected the top 150 up-regulated and down-regulated genes between two diseases for each RRHO analysis result and analyzed them with the Connectivity Map dataset on the cloud-based CLUE software platform (15) (version: 1.1.1.43, https://clue.io). This database allowed us to find compounds that drive down the input gene lists and identify potential drugs for treating one or more autoimmune diseases. Only classes with a median tau score < −85 were considered as potential target candidates.

## Results

### Transcriptomic Signatures in the Target Tissues of Different Autoimmune Diseases Reveal the Interaction Between Genetic and Environmental Factors

**Figure 1:**
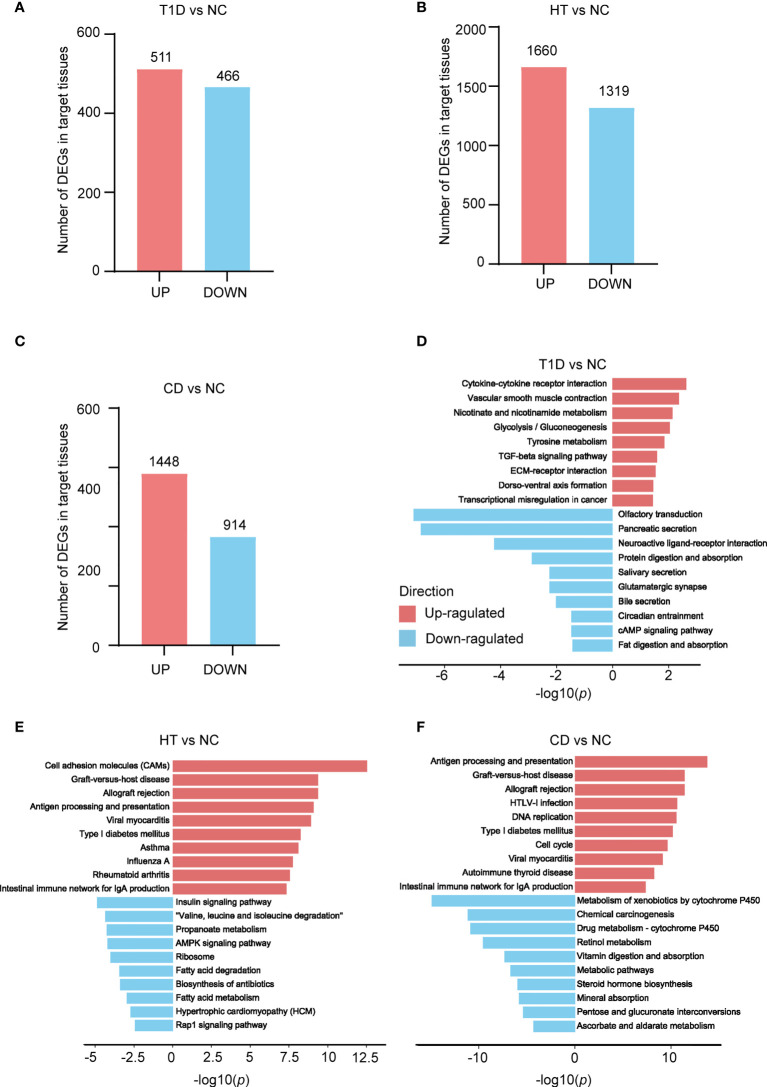
Depicts a summary of the count of differentially expressed genes (DEGs) and KEGG pathways found in the target organs of three autoimmune disorders. **(A–C)** The number of genes that differ in expression in three autoimmune diseases. The numbers within the bars indicate the count of genes with |fold change| greater than 1.5 and a *p* value less than 0.05. **(D–F)** KEGG enrichment analysis of T1D **(D)**, HT **(E)**, and CD **(F)** DEGs, in comparison to its own control set of individuals respectively. The red and blue bars indicate positive and negative enrichment in the associated pathway, respectively. The x axis represents the -log10 (p) of the enrichment analysis, and the y axis represents the enriched pathways.

To identify transcriptomic signatures in the target tissues of T1D, HT, and CD, differential expression analysis was performed in these three datasets respectively. The metadata of the tissue donors analyzed in the present study was shown in **Table 1**. In three datasets, there were more up-regulated DEGs than down-regulated DEGs in the target tissue, which was especially obvious in the CD dataset (**Figures 1A–C**). KEGG pathway enrichment analysis of these DEGs indicated similarities and differences between these three autoimmune diseases. The top up-regulated and down-regulated KEGG pathways were shown in **Figures 1D–F**. In the T1D dataset, up-regulated DEGs were primarily enriched in inflammation and metabolism, such as cytokine-cytokine receptor interaction, TGF-beta signaling pathway, and glycolysis/gluconeogenesis. Both up-regulated DEGs in HT and CD were enriched in autoimmunity and virus infection among the top 10 pathways, such as antigen processing and presentation, graft-versus-host disease, allograft rejection, type 1 diabetes, and intestinal immune network for IgA production, and viral myocarditis. All down-regulated DEGs in three datasets highlighted the metabolic related pathways, with protein digestion and absorption and fat digestion and absorption in the T1D dataset, fatty acid degradation and fatty acid metabolism in the HT dataset, and metabolic pathways in the CD dataset. In summary, differential expression analysis and enrichment analysis suggest autoimmunity, metabolism, and virus infection might be common pathways in these three diseases.

**Table 1:** Summary of the metadata for the microarray samples of the three autoimmune diseases.

Disease	Target tissue	Samples (n)		Age (mean ± SD)		Gender (Female%)		Nationality	Disease severity	Platforms	Experiment type	Source
		Controls	Patients	Controls	Patients	Controls	Patients					
T1D	Pancreatic tissue	7	10	24.57 ± 10.10	27.70 ± 7.23	100%	30%	USA	c-peptide (ng/ml):(NC:4.91 ± 2.86; T1D:0.36 ± 0.99)	GPL14550	Expression profiling by array	GSE72492
HT	Thyroid tissue	3	13	NA	47.85 ± 12.58	NA	92%	Germany	NA	GPL6244	Expression profiling by array	GSE138198
CD	Duodenal probes data	21	25	11.29 ± 4.91	9.28 ± 4.89	76%	60%	Germany	marsh stage:(NC:0-1; CD:3A-3C)	GPL10558	Expression profiling by array	GSE164883

Microarray data from three autoimmune disease studies of target tissues were obtained from the Gene Expression Omnibus (GEO) database (https://ncbi.nlm.nih.gov/geo/), reanalyzed, and quantified using the online tool NetworkAnalyst (https://www.networkanalyst.ca/). NA, not available. NC, normal control.

In order to explore the genetic impact on the target tissues, the risk genes of each disease were identified using the genome-wide association study (GWAS) catalog. Our results showed that 50-70 percent of these risk genes were expressed in target organs, while less than 30 percent of the risk genes overlapped with DEGs in each disease (**Figures S1A–C**). Overlapping of the risk genes of these three diseases revealed that only eight risk genes were in the intersection, namely, *ATXN2*, *ICOS*, *CTLA4*, *BACH2*, *HLA-DQA1*, *STAT4*, *SH2B3*, and *RNU6-474P*, among which *ICOS* and *HLA-DQAI* are DEGs in the three autoimmune diseases (**Figure S1D**). These findings indicate that other factors not only genetic factors play important roles in the occurrence and development of autoimmune diseases.

### RRHO Analysis of Autoimmune Diseases Indicates Up-Regulation of Viral Infection-Related Pathways

To study the common molecular mechanisms of these three autoimmune diseases in the target tissues, we analyzed the overlapping DEGs among these three datasets. However, there were only 17 overlapped DEGs, with 16 up-regulated genes (*CD38*, *IFI27*, *IFI16*, *XAF1*, *ITK*, *HLA-DQA1*, *MEI1*, *ICOS*, *GBP5*, *THEMIS*, *KLRB1*, *ANXA2R*, *GBP2*, *HK1*, *IRF8*, and *IL2RB*) and one down-regulated gene(*ACBD4* **Figures S2A–B**). These genes were mostly enriched in immune and inflammatory related pathways, such as the T cell receptor signaling pathway, immune response, IFN-γ signaling pathway, and type 1 interferon signaling pathway (**Figure S2C**). A limitation of this method is that we focused on the DEGs that pass a fixed statistical threshold, making the results greatly influenced by the number of samples studied.

To investigate if these common pathways were present before disease onset, 6 auto-antibody positive high risk individuals in the T1D dataset were used for functional enrichment analysis. The detailed clinical and HLA genotype information were presented in **Tables S1**, **S2**, which showed normal controls, auto-antibody positive individuals were well matched for HLA genotypes to the patients with T1D. Interestingly, autoimmune, metabolic, and virus infection related pathways were altered even in the auto-antibody positive phase (**Figures S3A–D**), implying the shared transcript footprint in autoimmune diseases exists before disease onset.

**Figure 2:**
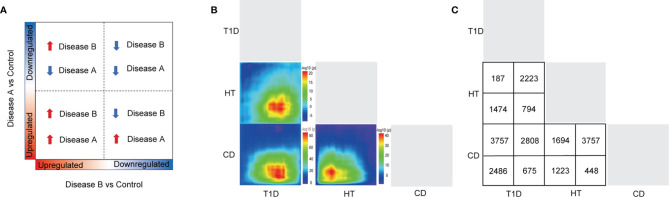
A RRHO analysis was carried out to compare target tissue gene expression features of three autoimmune disorders. **(A, B)** The RRHO algorithm was run on genes that were ranked by fold change from most down-regulated to most up-regulated. The level map colors represent the adjusted log *p* values of the overlap between genes(from red to blue, the adjusted log *p* values go from high to low) that are up-regulated in both disorders (bottom left quadrant), down-regulated in both disorders (top right quadrant), up-regulated in the left-hand labeled disease and down-regulated in the bottom labeled disease (top left quadrant), and down-regulated in the left-hand labeled disease and up-regulated in the bottom labeled disease (top left quadrant) (bottom right quadrant). **(C)** The number of genes that overlap in each pairwise analysis is shown in the panel.

**Figure 3:**
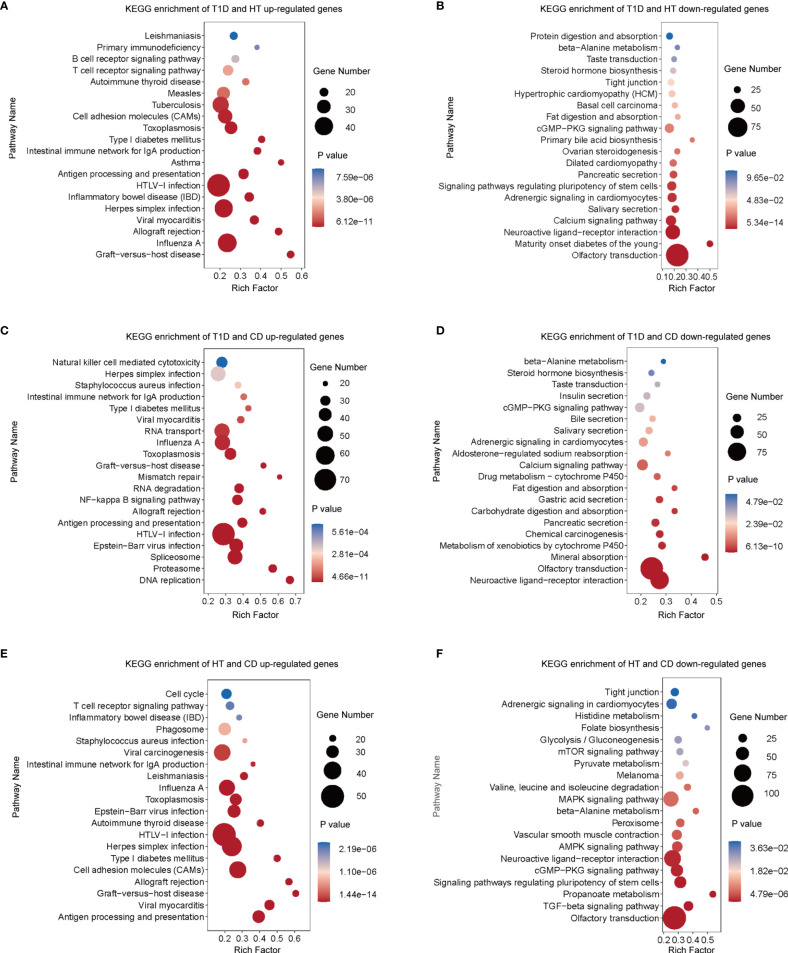
In three types of autoimmune disorders, functional enrichment analysis of overlapping genes revealed common signal pathways. **(A–F)** In the RRHO analysis (**Figures 2B**), genes with significant overlapping between different pairs of autoimmune diseases were chosen for enrichment analysis with the KEGG database. According to their *p* values, the top 20 gene sets are represented. Diseases were studied in pairs. **(A)** Up-regulated overlapping genes of T1D and HT. **(B)** Down-regulated overlapping genes of T1D and HT. **(C)** Up-regulated overlapping genes of T1D and CD. **(D)** Down-regulated overlapping genes of T1D and CD. **(E)** Up-regulated overlapping genes of HT and CD. **(F)** Down-regulated overlapping genes of HT and CD.

To achieve more detailed information on the similarities between different autoimmune diseases with an unbiased approach, we performed RRHO analysis (**Figures 2A–C**), which is a threshold-free algorithm that identifies trends of overlap between two ranked lists of genes according to fold change value. The main similarities between the diseases were observed both among the common up-regulated genes and down-regulated genes. Strikingly, the common upregulated genes between HT and CD had the highest logarithmic transformation’s hypergeometric *p* value. This finding is consistent with the above-described observation that up-regulated DEGs in HT and CD were enriched in several same pathways among the top 10 pathways. The KEGG pathway enrichment analysis of these up-regulated overlapping pathways demonstrated concordance for viral infection associated pathways (**Figures 3A, C, E**), including herpes simplex, HTLV-1, influenza A, and viral myocarditis. The common down-regulated genes were mainly involved in metabolism, including beta-alanine metabolism, adrenergic signaling in cardiomyocytes, cGMP-PKG signaling pathway, and neuroactive ligand-receptor (**Figures 3B, D, F**).

To further validate the shared pathways in the above RRHO analysis, we obtained a single cell RNA-seq data of islet samples of one patient with T1D and HT and one matched normal control. The detailed information of these data was presented in **Table S3**. Interestingly, the functional enrichment showed significantly upregulated in the viral gene expression pathway, which further verified our finding in microarray datasets (**Figure S4**).

To obtain more information on the shared pathways of these three autoimmune diseases, specific genes enriched in the enriched pathways were shown in **Table 2**. As expected, there were many HLA genes enriched in the shared autoimmune related pathways. Interestingly, virus infection associated pathways also contained a large number of HLA genes. Apart from this similarity, different virus infection pathways owned their unique genes, with *STAT1*,*RSAD2* and *IRF9* in the influenza A pathway,*IL-2RA*,*IL-2RB* and *CD40* in the HTLV-1 infection pathway, *HCFC1*, *MAP3K7*, *SOCS3*, *IRF9*, *HMGN1*, and *FAS* in the herpes simplex infection pathway. Collectively, altered gene expressions in the shared pathway of different autoimmune diseases might serve as diagnostic biomarkers for the occurrence and development of multiple autoimmune diseases.

**Table 2:** Common genes enriched in overlapping pathways in the RRHO results of three autoimmune diseases.

KEGG pathway	Gene symbol
Up regulated	
hsa04672: Intestinal immune network for IgA production	*HLA-DRA, CD40, HLA-DOB, HLA-DMA, HLA-DPB1, ICOS, HLA-DQA1, HLA-DPA1, PIGR, ITGB7, HLA-DMB, HLA-DQA2*
hsa05164: Influenza A	*PRKCB, CASP1, MAPK1, CIITA, TNFRSF10B, HLA-DPB1, STAT1, HLA-DQA1, PYCARD, ATF2, RSAD2, HLA-DRA, HLA-DMA, PIK3CD, MYD88, HLA-DPA1, ICAM1, HLA-DQA2, HLA-DOB, SOCS3, IRF9, ACTG1, FAS, HLA-DMB*
hsa05166: HTLV-I infection	*HLA-A, ETS1, CCND2, ANAPC7, IL2RB, ANAPC1, CHEK2, ADCY7, HLA-DPB1, HLA-DQA1, ATF2, LCK, HLA-DRA, HLA-DMA, PIK3CD, EGR1, EGR2, HLA-DPA1, ICAM1, HLA-DQA2, CD40, HLA-DOB, IL2RA, JAK3, HLA-E, MYB, HLA-DMB, HLA-G*
hsa05168: Herpes simplex infection	*HLA-A, SP100, HLA-DPB1, STAT1, HLA-DQA1, TRAF5, CD74, HLA-DRA, HLA-DMA, MYD88, HCFC1, HLA-DPA1, MAP3K7, HLA-DQA2, HLA-DOB, SOCS3, IRF9, HMGN1, HLA-E, FAS, HLA-DMB, HLA-G*
hsa04940: Type I diabetes mellitus	*HLA-DRA, HLA-DOB, HLA-A, HLA-DMA, HLA-DPB1, HLA-DQA1, HLA-DPA1, HLA-E, FAS, HLA-DMB, HLA-DQA2, HLA-G*
hsa05330: Allograft rejection	*HLA-DRA, CD40, HLA-DOB, HLA-A, HLA-DMA, HLA-DPB1, HLA-DQA1, HLA-DPA1, HLA-E, FAS, HLA-DMB, HLA-DQA2, HLA-G*
hsa05332: Graft-versus-host disease	*HLA-DRA, HLA-DOB, HLA-A, HLA-DMA, HLA-DPB1, HLA-DQA1, HLA-DPA1, HLA-E, FAS, HLA-DMB, HLA-DQA2, HLA-G*
hsa05416: Viral myocarditis	*HLA-DRA, HLA-A, HLA-DMA, HLA-DPA1, FYN, ICAM1, HLA-DQA2, CD40, HLA-DOB, HLA-DPB1, HLA-DQA1, HLA-E, ACTG1, HLA-DMB, HLA-G*
Down regulated	
hsa04261: Adrenergic signaling in cardiomyocytes	*AGTR2, MYH6, PPP2R2D, CACNG4, ADRB1, CACNG6, SCN5A, ATP2B2, KCNE1, ADRA1D, CACNA1S*
hsa00410: beta-Alanine metabolism	*SMOX, ALDH3A1, ALDH3B1, DPYS, GADL1, HIBCH, UPB1*
hsa04080: Neuroactive ligand-receptor interaction	*NPY5R, GRIN1, PLG, NMBR, ADRA2C, GRIA4, TACR3, GRM1, GRM7, GRM5, P2RY1, ADRB1, OPRD1, CHRM5, ADRA1D, TAAR2, MCHR2, HRH3, GH2, AGTR2, OPRK1, HRH1, GHRHR, GRIK1, NPFFR1, NTSR2, PRLR, GRM4, P2RY2, GLRA3, FSHR, MCHR1, THRA, OPRM1*
hsa04022: cGMP-PKG signaling pathway	*ATP2A1, ADRA2C, IRS4, MYLK4, ADRB1, MYLK3, CNGB1, INS, OPRD1, GUCY1A2, ATP2B2, ADRA1D, MYLK2, GTF2IRD1, GUCY1B3, CACNA1S*

The RRHO analysis results were selected to enrich the overlapping KEGG pathways in the analysis, and the intersection of genes corresponding to a single pathway in each RRHO analysis list was taken, with the results shown in the table.

### The Inhibitors of Tyrosine Kinase, Phosphoinositide 3-Kinase, and Heat Shock Protein May Be Potential Therapeutic Targets in Autoimmune Diseases

**Figure 4:**
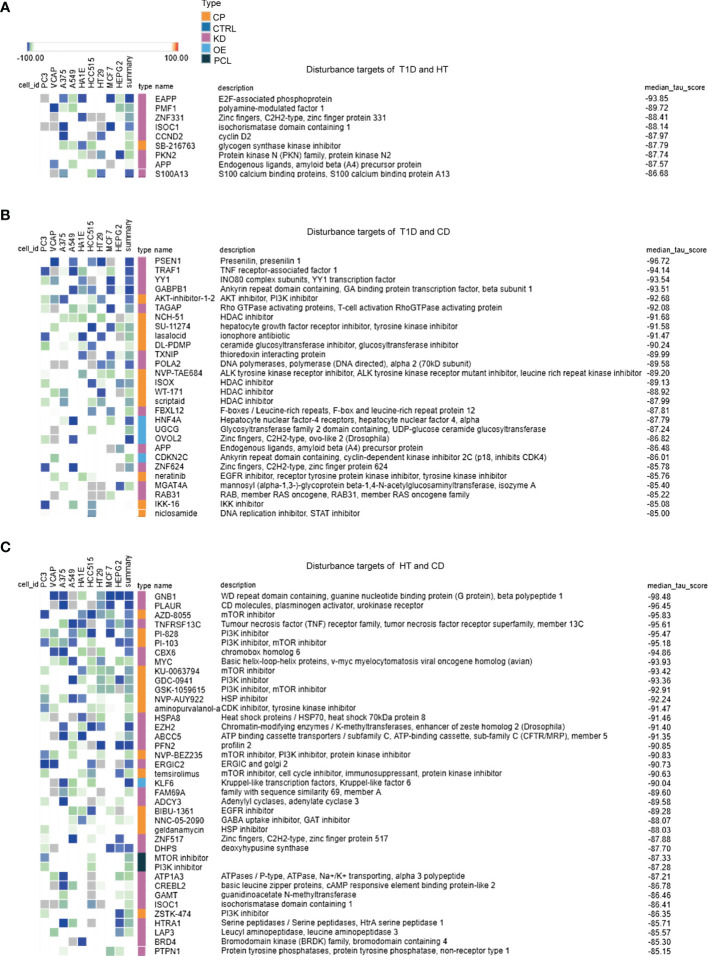
Exploration of overlapping genes among target tissues in three autoimmune disorders leads to the discovery of common therapeutic targets. **(A–C)** For each RRHO analysis, the top 150 up or down overlapping genes were submitted to the Connectivity Map database in order to identify perturbagen classes that cause an opposite effect (negative tau score) in the target tissues of autoimmune diseases. Only classes with a median tau score < -85 were represented. Perturbagen classes cause an opposite effect in the genomic signatures of up and down overlapping genes. The above methodology and conditions were used for the following analysis: **(A)** T1D and HT, **(B)** T1D and CD, **(C)** HT and CD.

Based on the overlapping genes and pathways in the target tissues, we were able to discover common therapeutic targets for multiple autoimmune diseases (**Figures 4A–C**). A large number of potential drug targets were indicated for patients with HT and CD, which was in line with our observations in the RRHO analysis. These results further indicated a closer relationship in the pathogenesis between HT and CD. There were fewer drugs or targets for patients with T1D and HT (**Figure 4A**), and the median tau score was lower. However, epidemiology revealed that HT and T1D were prone to co-occurrence (2), suggesting that more research was required to investigate the common mechanisms and therapeutic targets. Tyrosine kinases inhibitors were predicted to treat T1D, HT, and CD (**Figures 4A–C**). In addition, PI3K inhibitors were potential drugs for patients with CD and T1D or patients with CD and HT (**Figures 4B, C**). Several novel targets, such as the HSP inhibitors, HDAC inhibitors, S100A family, and zinc fingers family might be therapeutic targets for multiple autoimmune diseases. In summary, a number of virus infections associated targets may be potential therapeutic targets in autoimmune diseases.

To further determine the optimal time for treatment, we also analyzed the autoantibody positive individual in the T1D dataset using RRHO and cMAP methods. Virus infection associated pathways were significantly enriched in overlapped upregulated genes between T1D and antibody positive individuals, such as human T-cell leukemia virus 1 infection, Epstein-Barr virus infection, and salmonella infection (**Figures S5A–D**).HDAC inhibitors were predicted to be potential targets for T1D and antibody positive individuals (**Figure S6**).These findings suggest that targeting the shared pathways in autoimmune diseases are recommended during the original viral infection in patients with more than one autoimmune disease, even in individuals at high risk. Indeed, there are some ongoing trials focusing on virus vaccines, which might be potential therapies for the prevention of autoimmune diseases (16).

## Discussion

In the present study, we discovered that different target tissues from three distinct autoimmune disorders, including T1D, HT, and CD, were affected by common environmental factors such as influenza A, HTLV-I, and herpes simplex infection. These data highlight the role of shared environmental factors in the pathogenesis of T1D, HT, and CD. Furthermore, our findings may provide novel biomarkers to aid the diagnosis as well as develop new strategies for the treatment of these autoimmune diseases.

The transcriptional profiling of the target tissues in T1D, HT, and CD showed up-regulation of autoimmunity-related pathways, such as intestinal immune network for IgA production, T1D, allograft rejection, and graft-versus-host disease. Current methods for comparing expression profiles typically involve selecting a fixed differential expression threshold to summarize results, potentially decreasing sensitivity to small but concordant changes. RRHO analysis is a method for detecting and visualizing overlap trends between two complete, continuous gene-expression profiles, which is unbiased, sensitive, robust, and web-accessible. In this study, we observed varying degrees of overlap between the diseases in terms of both up-regulated and down-regulated genes, particularly the up-regulated expression patterns between HT and CD.

Interestingly, these three organ-specific autoimmune diseases all demonstrated consistently up-regulated pathways associated with virus infection, including influenza A, HTLV-1 infection, herpes simplex infection, and viral myocarditis. Virological evidence for T1D, HT, and CD are at different levels, with a majority of serological data or studies on circulating viruses, some epidemiological data, and few studies on direct proof of target tissue infection (17–19). In the subgroup with laboratory-confirmed pandemic influenza A, a nationwide cohort study found a twofold increase in the incidence of T1D (20). Another national cohort study reported that individuals with CD have a higher probability of hospital admission for influenza (21). According to many case reports, these four viruses were associated with T1D, HT, and CD (22). However, no research has looked into the connection between the virus infection and the occurrence of T1D, HT, and CD in the same patient. Our findings imply that these viruses are common environmental triggers in the pathogenesis of T1D, HT, and CD. In medical practice, taking virus infection histories is required (23). Individuals at high risk for multiple autoimmune diseases should be examined for antibodies targeting influenza A, HTLV-I infection, and herpes simplex infection as needed. The results of this study indicate that virus infections may play the same important role as genetic background in the etiology of T1D, HT, and CD. However, the mechanisms through which viruses cause autoimmune disorders are unknown. Influenza A induces toll-like receptor 3 overexpression in thyrocytes, which are associated with HT (24). Several epidemiological studies in human and animal experiments have demonstrated that virus infections can either trigger or prevent autoimmune pathologies, depending on various factors such as genetic background, the onset time of infection, viral load, type of virus strain, and host-elicited immune responses. Nonetheless, few studies have focused on the specific mechanistic interaction between the virus and the immune system in the process of autoreactivity. Previous studies showed that virus-induced autoimmunity can be activated by various mechanisms including bystander activation, epitope spreading, molecular mimicry, and immortalization of infected B cells (25). The molecular mechanisms underlying the potential etiological relationship between viruses and autoimmune diseases should be investigated.

Because of the observed similarities in pathway activation between target tissues, several classes of drugs that could potentially be used to treat more than one autoimmune disease were identified. The tyrosine kinase inhibitor is of particular interest among them. Tyrosine kinases are important signaling cascade mediators, playing critical roles in a variety of biological processes such as growth, differentiation, apoptosis, and metabolism in response to internal and external stimuli. It is worth noting that imatinib, a tyrosine kinase inhibitor, was shown in a multicenter, randomized, double-blind, placebo-controlled, phase 2 trial to preserve beta-cell function in patients with recent-onset T1D (26). Possible mechanisms of imatinib might include, 1) inhibiting ABL-IRE1α interaction and dampening IRE1α RNase hyperactivity, thus resulting in the reduction of pancreatic β cell apoptosis as demonstrated in a non-obese diabetic (NOD) mouse study (27); 2) preventing the process of T cell and macrophage infiltration into islets (28). Notably, tyrosine kinase inhibitors exhibit potent antiviral activity by blocking multiple steps of influenza A virus replication (29). Collectively, tyrosine kinase inhibitors are entering the clinical research stage for T1D patients. Our study showed that tyrosine kinase inhibitors could also be potential drugs for HT and CD.

Another class of drug identified in this study for potential use in multiple autoimmune diseases is HSP inhibitors. Although no HSP inhibitor-related studies in T1D, HT, or CD have been conducted, it has been extensively documented that HSP70 triggers the autoimmune disease. HSP70 promotes the function of antigen-presenting cells (dendritic cells) and converts T-cell tolerance to autoimmunity (30). Higher expression of HSP70 was shown in patients with T1D or celiac disease (31, 32). In a large cohort of T1D subjects, serum anti-HSP70 antibody levels were independently and inversely related to diabetic vascular complications, implying that anti-HSP70 antibody levels may be a potential marker of protection from diabetic vascular complications (33). HSP70 has also been reported to modulate the activity of influenza a virus polymerase (34). HSP70 may prime protective immune responses to herpes viruses early in life during infection through binding recombinant viral protein and viral epitopes, which can be potentially lethal, and the establishment of latency frequently occurs (35). Taken together, previous studies showed that HSP is closely associated with chronic diabetic complications of T1D and virus infections, and our analysis revealed that HSP inhibitors might be potential drugs for HT and CD. All the above results suggest that HSP inhibitors may be novel drugs for treating multiple autoimmune diseases by regulating immunity and defending against virus infections.

PI3K inhibitors were another potential treatment for multiple autoimmune diseases. These compounds inhibit a group of lipid kinases, which phosphorylates phosphoinositide from cell membranes, regulating cellular processes such as immune responses, cell growth, and metabolism. PI3K inhibition could be a novel therapeutic approach for treating vascular dysfunction in patients with diabetes (36). It was reported that NOD mice with PI3Kγ deficiency were protected from developing spontaneous diabetes. Moreover, a recent study showed that PI3K-inhibitor suppressed the proliferation and cytokine production of a human CD4^+^ T-cell clone specific for GAD peptide isolated from a T1D patient (37). In agreement with our study, these findings suggest an involvement of the PI3K pathway in the regulation of autoimmune diabetes and provide rationales for the future use of anti-PI3K therapy in T1D. Furthermore, PI3K/Akt expression increased in HT patients, indicating a possible molecular mechanism of PI3K/Akt in the pathogenesis of HT (38). However, more research focusing on PI3K inhibitors for multiple autoimmune diseases is required.

Several limitations to this study need to be acknowledged. First, the sample numbers of the resource gene data were relatively small, therefore, further study on large scale is required to validate these findings. Second, the resource gene data were from the comparison of patients with a single disease and controls, more samples from patients with two or three diseases of T1D, HT, and CD are required to validate the shared genetic fingerprint. Third, more in-vivo and in-vitro researches are needed to better understand the precise molecular mechanism, providing new targets for disease prevention and therapy.

It is well recognized that autoimmune diseases are caused by a complex interaction between genetic and environmental factors. Our combination analysis of GWAS data and gene signatures of different target organs provide more reliable evidence demonstrating that virus infection, especially influenza A, HTLV-I, and herpes simplex infection, may be critical environmental factors in triggering diseases in genetically susceptible individuals, as more than 70 percent of the candidate GWAS genes were not in the DEGs of T1D, HT, and CD. Tyrosine kinase inhibitors, HSP inhibitors, and PI3K inhibitors could be potential novel treatments for multiple autoimmune diseases. Collectively, we deem that our findings contribute to a better understanding of the pathogenesis of T1D, HT, and CD, which might provide new insights for identifying novel biomarkers for the diagnosis and new targets for the treatment of these diseases.

## Data Availability Statement

The datasets presented in this study can be found in online repositories. The names of the repository/repositories and accession number(s) can be found in the article/**Supplementary Material**.

## Author Contributions

MY designed the experiment, performed the data analysis and drafted the manuscript. YZ performed the data analysis and drafted the manuscript. SL revised the manuscript. XL and JH designed the experiment and revised the manuscript. All authors read and approved the final manuscript. All authors contributed to the article and approved the submitted version.

## Funding

This study was supported by the National Natural Science Foundation of China (grant no. 82070812), the Science and Technology Innovation Program of Hunan Province (2020RC4044), the Hunan Provincial Innovation Foundation for Postgraduate (grant no. CX20210363) and the Fundamental Research Funds for the Central Universities of Central South University (grant no. 2021zzts0361).

## Conflict of Interest

The authors declare that the research was conducted in the absence of any commercial or financial relationships that could be construed as a potential conflict of interest.

## Publisher’s Note

All claims expressed in this article are solely those of the authors and do not necessarily represent those of their affiliated organizations, or those of the publisher, the editors and the reviewers. Any product that may be evaluated in this article, or claim that may be made by its manufacturer, is not guaranteed or endorsed by the publisher.
