# Supplementary material for: Gene Expression Signatures Reveal Common Virus Infection Pathways in Target Tissues of Type 1 Diabetes, Hashimoto’s Thyroiditis, and Celiac Disease
Source: Front Immunol. 2022 Jun 20;13:891698. doi: 10.3389/fimmu.2022.891698 (PMC9251511; doi:10.3389/fimmu.2022.891698)
Supplement: Supplementary file 1 [file DataSheet_1.docx]

Figure S1


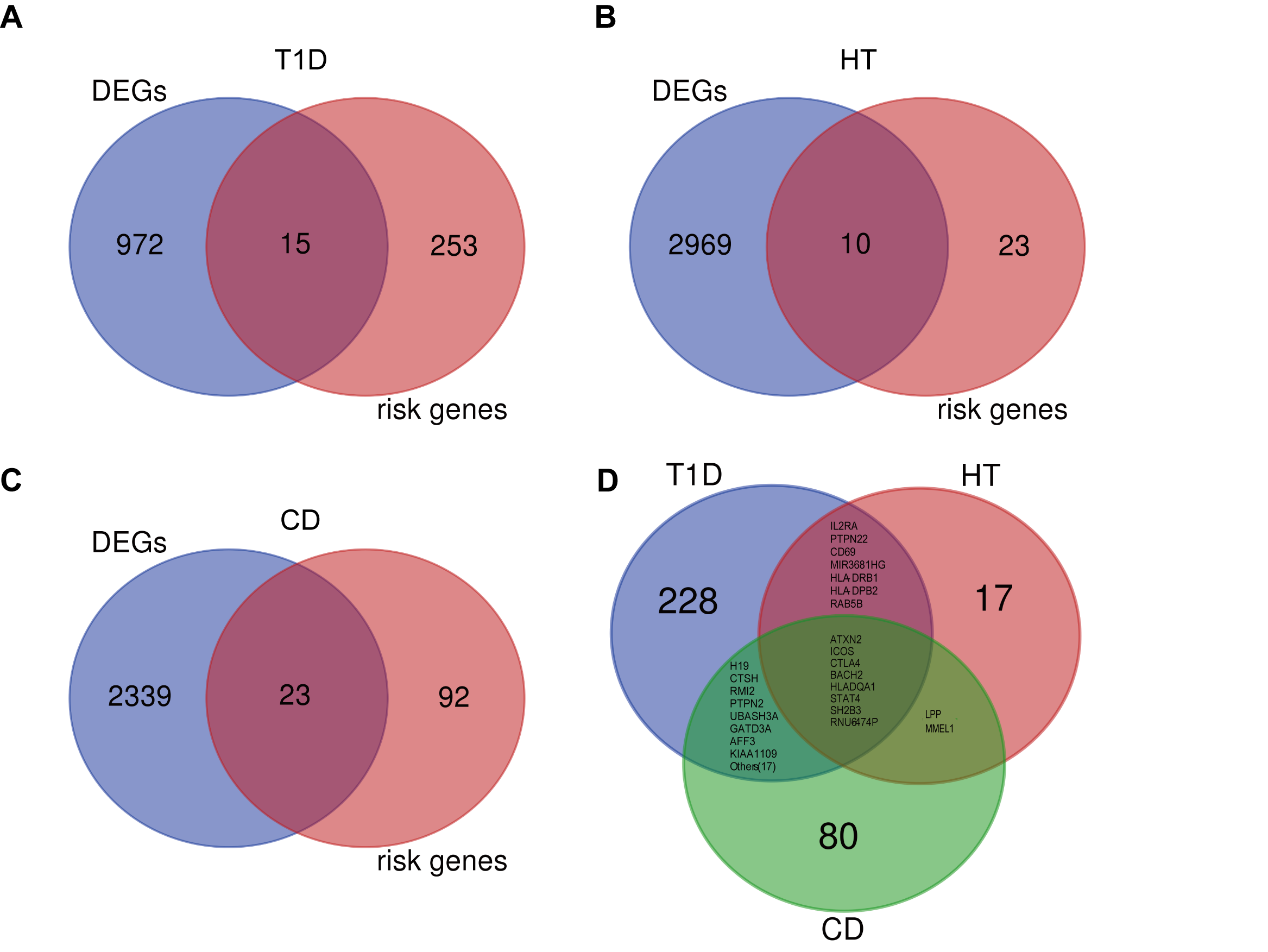


Figure S2


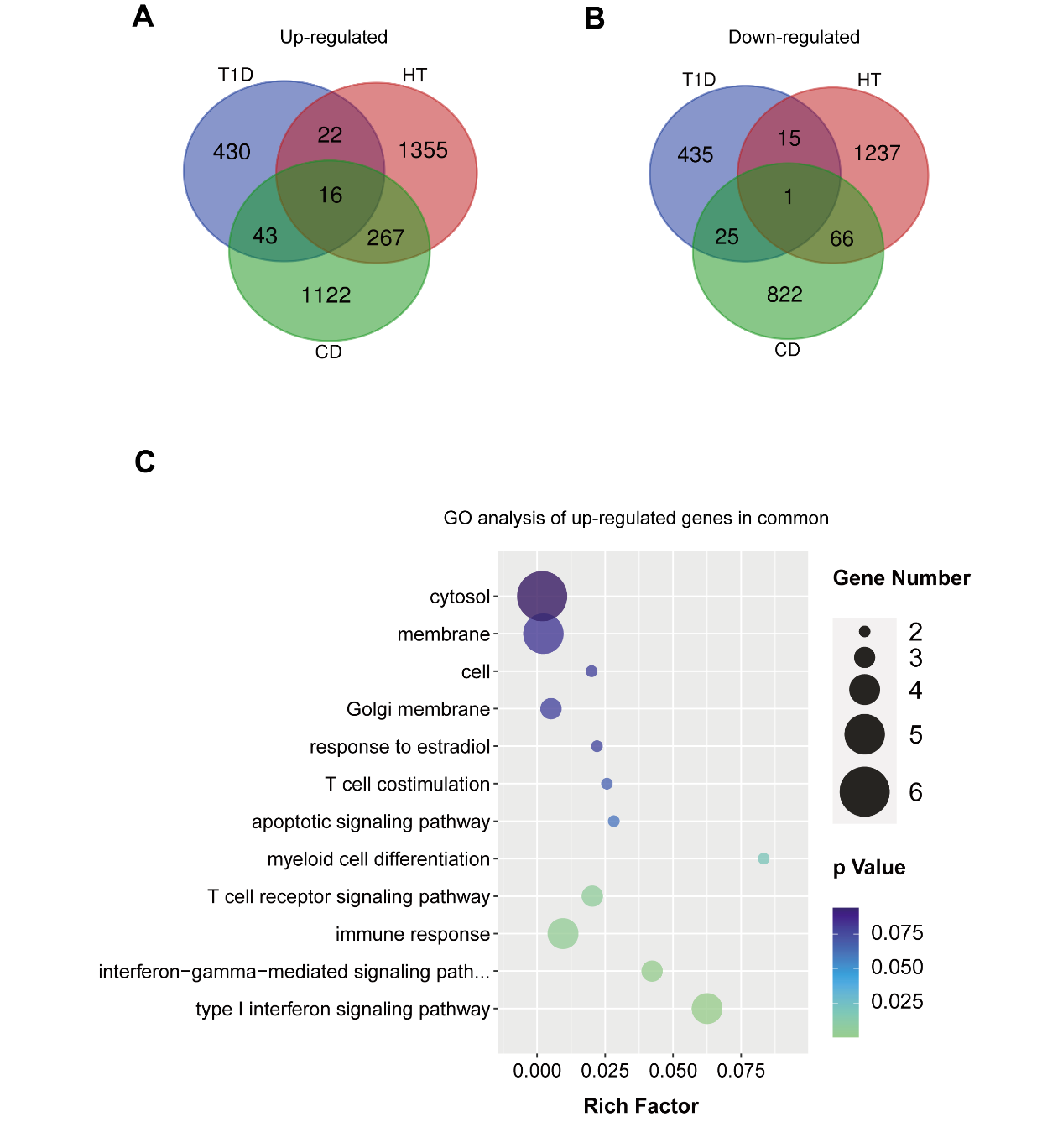


Figure S3


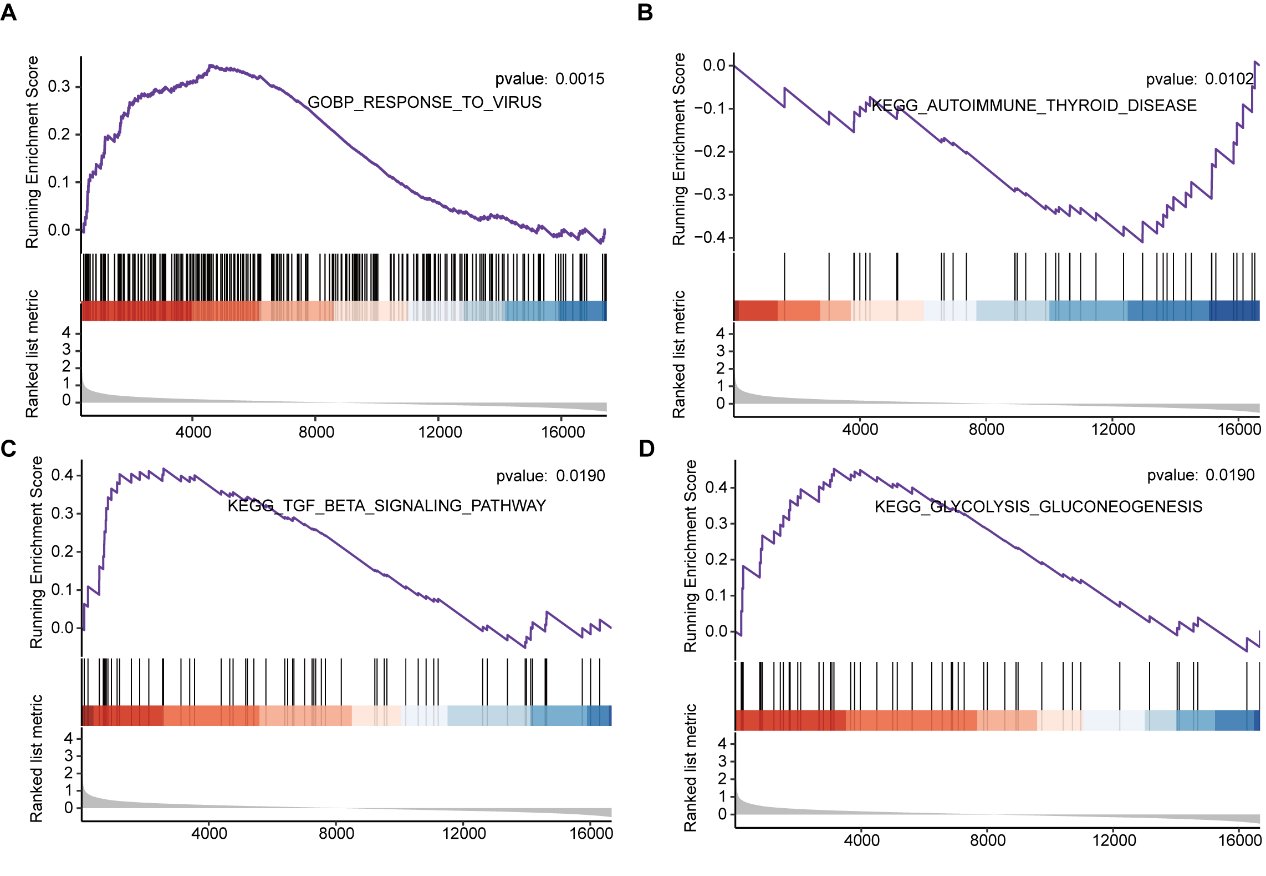


Figure S4


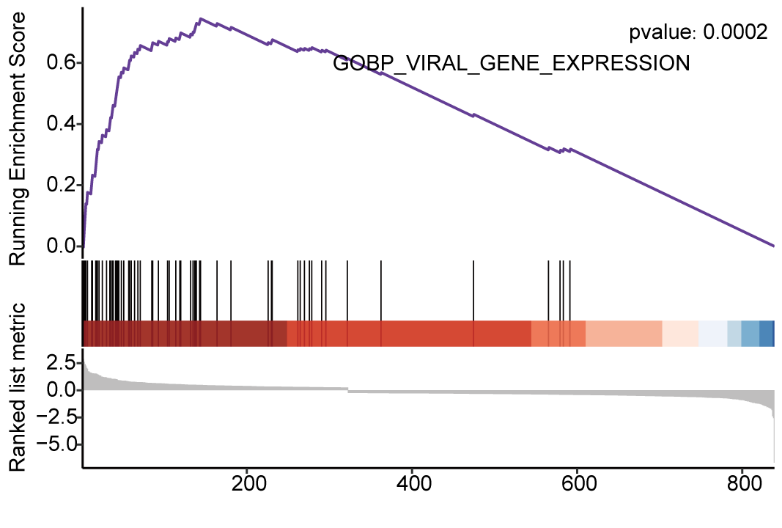


Figure S5


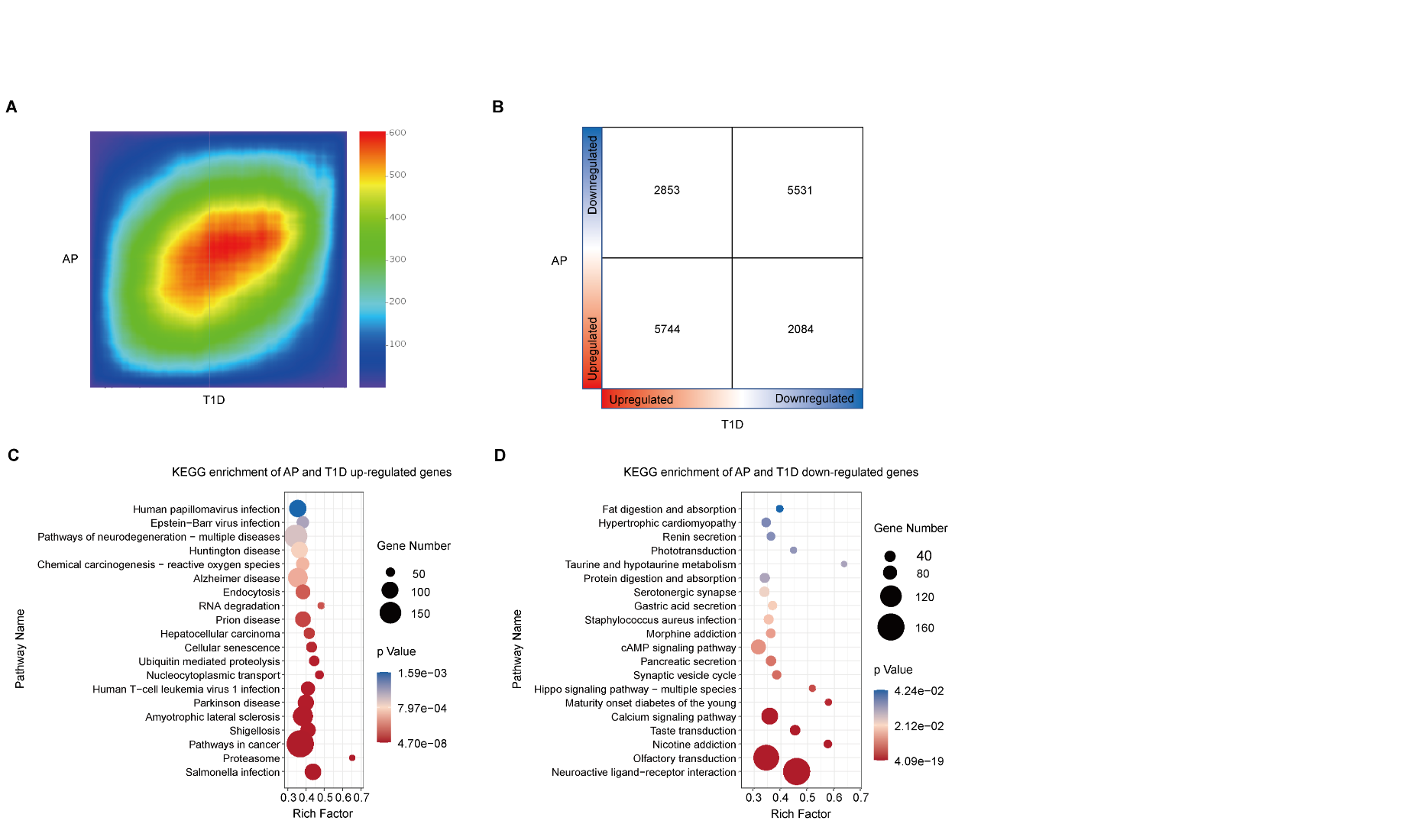


Figure S6


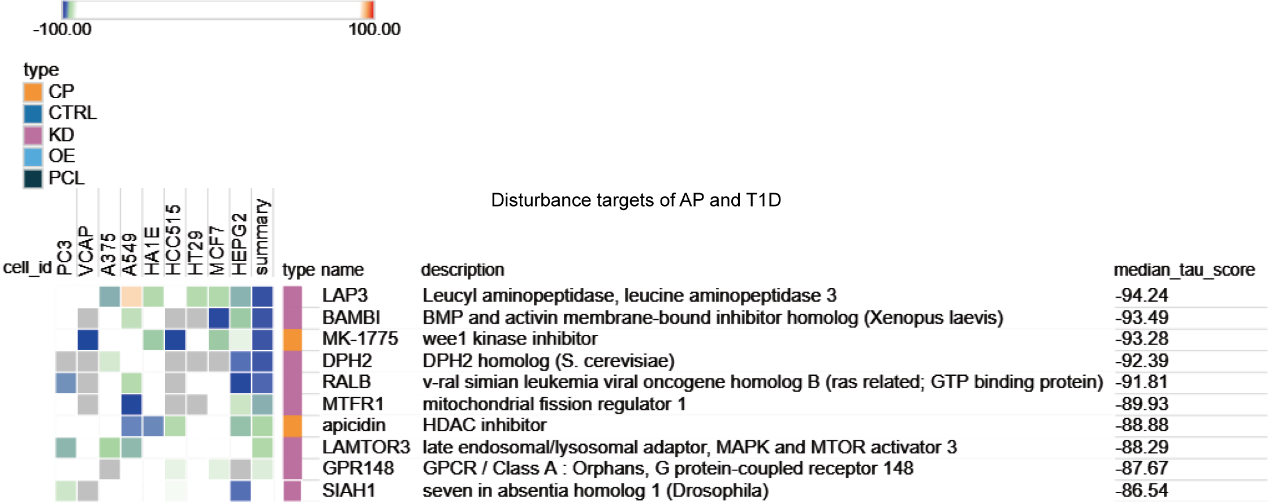


**Fig. S1.** Transcriptome effects of risk genes in three autoimmune disease target organs. (A) Overlap of risk genes and DEGs in T1D; (B) Overlap of risk genes and DEGs in HT; (C) Overlap of risk genes and DEGs in CD; (D) Overlap of risk genes in three diseases.

**Fig. S2.** Traditional methods yielded common differential genes in autoimmune disease target tissues. (A) Up-regulated Venn diagram of gene intersection. (B) Down-regulated Venn diagram of gene intersection (right) in three data sets. (C) GO analysis of 16 up-regulated genes in common.

**Fig. S3.** The transcriptome of subjects with auto-antibody positive but not diagnosed with type 1 diabetes (AP) phase demonstrated an increase in the expression of virus-related pathways. (A-D) GSEA enrichment results of genetic difference analysis of AP data versus normal controls.

**Fig. S4.** GSEA enrichment analysis of single cell sequencing revealed that virus-associated pathway was up-regulated in type 1 diabetes (T1D) with Hashimoto's thyroiditis (HT).

**Fig. S5**. RRHO revealed common features of the transcriptome in both auto-antibody positive but not diagnosed with type 1 diabetes (AP) individuals and type 1 diabetes (T1D) patients. (A-B) RRHO analysis between AP and T1D. (C) KEGG enrichment of overlapping upregulated genes observed in RRHO analysis of AP and T1D. (D) KEGG enrichment of overlapping downregulated genes was observed in RRHO analysis of AP and T1D.

**Fig. S6.** Exploration of overlapping genes between auto-antibody positive but not diagnosed with type 1 diabetes (AP) and type 1 diabetes (T1D) leads to the discovery of common therapeutic targets.

Table S1. Summary of the metadata for the auto-antibody positive but not diagnosed with type 1 diabetes (AP) samples.

| **Disease** | **Target tissue** | **Samples (*n*)** | | **Age (years)** | | **Gender (Female%)** | | **Nation** | **Severity** | **Platforms** | **Experiment type** | **Source** |  |
| --- | --- | --- | --- | --- | --- | --- | --- | --- | --- | --- | --- | --- | --- |
|  |  | **Controls** | **Patients** | **Controls** | **Patients** | **Controls** | **Patients** |  |  |  |  |  |  |
| AP | Pancreatic tissue | 7 | 10 | 24.57±10.10 | 24.33±15.19 | 100% | 50% | USA | c-peptide (ng/ml):(NC: 4.91±2.86; AP：6.51±6.25) | GPL14550 | Expression profiling by array | GSE72492 |  |
|  |  |  |  |  |  |  |  |  |  |  |  |  |  |

Table S2. HLA genotypes of each group in GSE72492

|  | NC (n = 2) | AP (n = 6) | T1D (n = 5) |
| --- | --- | --- | --- |
| DR3DR4 (%) | 50 (1) | 0 | 40 (2) |
| DR3/DR4 (%) | 0 | 83.3 (5) | 60 (3) |
| DR3 (%) | 0 | 33.3 (2) | 40 (2) |
| DR4 (%) | 0 | 50 (3) | 20 (1) |
| DRX (%) | 50 (1) | 16.7 (1) | 0 |

Note: Not all samples were tested for HLA genotypes, only the data provided by the authors are presented in Table S2.

Table S3. Summary of the metadata for the type 1 diabetes (T1D) with Hashimoto's thyroiditis (HT) samples.

| **Disease** | **Target tissue** | **Samples (*n*)** | | **Age (years)** | | **Gender (Female%)** | | **Nation** | **Severity** | **Experiment type** | **Source** |
| --- | --- | --- | --- | --- | --- | --- | --- | --- | --- | --- | --- |
|  |  | **Controls** | **Patients** | **Controls** | **Patients** | **Controls** | **Patients** |  |  |  |  |
| T1D & HT | pancreas islet | 1 | 1 | 5 | 10 | 100% | 100% | USA | c-peptide (ng/ml): (NC:1.88; T1D & HT: 0.02) | Single cell sequencing | HPAP-039 (NC), HPAP-032 (T1D & HT) |
